# Obesity Worsens Gulf War Illness Symptom Persistence Pathology by Linking Altered Gut Microbiome Species to Long-Term Gastrointestinal, Hepatic, and Neuronal Inflammation in a Mouse Model

**DOI:** 10.3390/nu12092764

**Published:** 2020-09-10

**Authors:** Dipro Bose, Punnag Saha, Ayan Mondal, Brian Fanelli, Ratanesh K. Seth, Patricia Janulewicz, Kimberly Sullivan, Stephen Lasley, Ronnie Horner, Rita R. Colwell, Ashok K Shetty, Nancy Klimas, Saurabh Chatterjee

**Affiliations:** 1 Environmental Health and Disease Laboratory, Department of Environmental Health Sciences, University of South Carolina, Columbia, SC 29208, USA; bosed@email.sc.edu (D.B.); psaha@email.sc.edu (P.S.); mondala@mailbox.sc.edu (A.M.); sethr@mailbox.sc.edu (R.K.S.); 2 Cosmos ID, Inc., Rockville, MD 20850, USA; brian.fanelli@cosmosid.com (B.F.); rita.colwell@cosmosid.com (R.R.C.); 3 Department of Environmental Health, Boston University School of Public Health, Boston, MA 02118, USA; paj@bu.edu (P.J.); tty@bu.edu (K.S.); 4 Department of Cancer Biology and Pharmacology, University of Illinois College of Medicine at Peoria, Peoria, IL 61605, USA; sml@uic.edu; 5 Department of Health Services Policy and Management, University of South Carolina, Columbia, SC 29208, USA; hornerrd@mailbox.sc.edu; 6 Institute for Regenerative Medicine, Department of Molecular and Cellular Medicine, Texas A&M Health Science Center College of Medicine, Temple and College Station, Bryan, TX 77842, USA; akskrs@tamu.edu; 7 Department of Clinical Immunology, Nova Southeastern University, Fort Lauderdale, FL 33314, USA; nklimas@nova.edu; 8 Miami VA Medical Center, Miami, FL 33125, USA

**Keywords:** dysbiosis, Western diet, metagenomics, bacterial species, whole genome sequencing, neuroinflammation, peroxynitrite, symptom persistence

## Abstract

Persistence of Gulf War illness (GWI) pathology among deployed veterans is a clinical challenge even after almost three decades. Recent studies show a higher prevalence of obesity and metabolic disturbances among Gulf War veterans primarily due to the existence of post-traumatic stress disorder (PTSD), chronic fatigue, sedentary lifestyle, and consumption of a high-carbohydrate/high-fat diet. We test the hypothesis that obesity from a Western-style diet alters host gut microbial species and worsens gastrointestinal and neuroinflammatory symptom persistence. We used a 5 month Western diet feeding in mice that received prior Gulf War (GW) chemical exposure to mimic the home phase obese phenotype of the deployed GW veterans. The host microbial profile in the Western diet-fed GWI mice showed a significant decrease in butyrogenic and immune health-restoring bacteria. The altered microbiome was associated with increased levels of IL6 in the serum, Claudin-2, IL6, and IL1β in the distal intestine with concurrent inflammatory lesions in the liver and hyperinsulinemia. Microbial dysbiosis was also associated with frontal cortex levels of increased IL6 and IL1β, activated microglia, decreased levels of brain derived neurotrophic factor (BDNF), and higher accumulation of phosphorylated Tau, an indicator of neuroinflammation-led increased risk of cognitive deficiencies. Mechanistically, serum from Western diet-fed mice with GWI significantly increased microglial activation in transformed microglial cells, increased tyrosyl radicals, and secreted IL6. Collectively, the results suggest that an existing obese phenotype in GWI worsens persistent gastrointestinal and neuronal inflammation, which may contribute to poor outcomes in restoring cognitive function and resolving fatigue, leading to the deterioration of quality of life.

## 1. Introduction

Persistence of Gulf War illness (GWI) among deployed veterans is a clinical challenge to diagnose and treat. Though there is strong epidemiological evidence of an association of Gulf War exposures to chronic fatigue, metabolic syndrome, gastrointestinal (GI) disturbances and obesity, the persistence or worsening of the symptoms even long after the war has ended and our troops have returned home remains a challenge for clinicians in numerous health centers nationwide [1,2,3,4,5]. A recent epidemiological study highlighted that overweight and obesity are highly prevalent among Gulf War and Gulf Era veterans [6,7]. Persistence of symptoms in ~47% of examined Gulf War (GW) veterans was linked to obesity [7,8]. There was also a strong association of post-traumatic stress disorder (PTSD) in GW veterans with metabolic syndrome and obesity [7]. The study implied that advancing age and lifestyle factors (Western diet, physical inactivity) are augmenting the development of obesity, metabolic syndrome, and other chronic diseases. It is interesting to note that PTSD can lead to physical inactivity and may increase this risk in aging veterans to develop age-related diseases [7]. Interestingly, a recent preliminary report in mouse models of GWI showed that a high-fat diet leads to dysbiosis, with a different microbiome signature than GWI mice fed a standard chow diet [9]. With the passage of 28 years since the GW, and the GW veterans reaching the age range of 50–60 years, physical inactivity/weight gain would likely lead to longstanding and exacerbated pathological changes in the brain, liver, and the gastrointestinal (GI) tract. A recent longitudinal study suggests that there is an immediate need to consider the relationship between persistently increasing symptoms and long-term morbidity, especially as veterans age [1]. The carefully designed longitudinal study showed that chronic fatigue (likely from metabolic dysfunction/mitochondrial dysfunction), headaches and pain (as a consequence of inflammation), flatulence or burping (due to GI disturbances), and loss of concentration (owing to neuronal dysfunction) increased or persisted in GW veterans 20 years after their return. 

In agreement with the above findings, the preclinical data demonstrated that mice exposed to pyridostigmine bromide and permethrin (PB + PER) exhibited persistent neurobehavioral deficits and neuroinflammation until 22.5 weeks post-exposure [5]. Notably, the adverse health effects of chemicals such as PER and chlorpyrifos associated with GWI also showed an association with obesity and metabolic complications [10]. In a recent study, we have shown that persistence of GWI even after 5 months of exposure largely depends on an altered microbiome and its association with increased damage-associated molecular pattern release from the GI tract, especially the epithelial cells [11]. We have also shown that the enteric glial cell activation resulting from of a sustained microbial dysbiosis in the gut plays a significant role in the release of damage-associated molecular patterns (DAMPs) that further influence an exacerbated neuroimmune response [12]. Interestingly, intestinal inflammation associated with irritable bowel syndrome (IBS), alcoholic steatohepatitis, obesity, non-alcoholic fatty liver disease, or metabolic syndrome has been strongly linked to alterations in the gut microbiome [13,14,15,16,17,18,19,20]. There are at least 3000 species of bacteria residing in the human gut, and there is a unique prototype of bacterial diversity in every individual [21]. The last decade saw a tremendous increase in our understanding of how gut bacteria participate in health and disease. Significant disorders where gut bacteria have been found to play a role include obesity, metabolic syndrome, and IBS [21]. 

GWI is also characterized by neuroinflammation and systemic inflammation with elevated levels of pro-inflammatory cytokines, including TNF-α and IL1β [22,23,24,25]. Recent literature shows important connections between microbiome, obesity, and central nervous system disorders with gut microbiome referred to as “second brain” [26,27,28,29]. Interestingly, IBS and obesity have a significant proinflammatory component that includes macrophage activation and triggering of toll-like receptor pathway [30,31]. Several studies have found strong evidence of a decrease in expression of gut junction proteins, leading to portal endotoxemia [32,33,34,35]. With dysbiosis, obesity, and metabolic syndrome being closely associated, it is not uncommon to find a low systemic inflammation causing significant modulation of gut-immune axis. Since a significant number of veterans presenting persistent GWI symptoms are also obese, it is likely that an underlying inflammatory condition due to obesity or metabolic syndrome contributes to the persistence and exacerbation of symptoms. However, the mechanisms by which obesity or metabolic syndrome leads to persistence of the GWI symptoms remain unclear. Thus, a significant knowledge gap exists about the relationship between GW chemical exposures, weight gain, metabolic syndrome, GI disturbances, and neuroinflammation among GW veterans, especially concerning an altered microbiome. Therefore, the present study investigated the combined effects of GW exposures and the consumption of the Western diet. Such a study is germane to a significant percentage of veterans with GWI because veterans have aged since the exposure to GW chemicals in the 1990s, are inactive physically (owing to incidences of PTSD, disabilities, and chronic fatigue) and likely to consume a standard American Diet (i.e., the Western diet, rich in fat and carbohydrates) [36]. The study addresses the paradigm that obesity potentiates persistence of GW chemical exposure-induced GI disturbances marked by alterations in tight junction protein levels, site-specific inflammation, metabolic syndrome, and neuroinflammation. Using an established mouse model of chronic GWI where animals received a Western diet for 5 months after GW chemical exposure, and shotgun metagenomics, we show that the alterations of species-specific microbiome persisted and worsened with obesity. The persistent microbiome alterations were strongly associated with GI inflammation, higher serum levels of proinflammatory levels of IL6, and insulin, which caused liver inflammation. Western diet-induced obesity was also associated with microglial activation that led to persistent neuroinflammation—a pathology correlated with altered microbiome.

## 2. Materials and Methods

### 2.1. Materials

Pyridostigmine bromide (PB) and permethrin (Per) were purchased from Sigma-Aldrich (St. Louis, MO, USA). Primary antibodies including anti-Claudin-2, anti-Occludin, anti-CD68, anti-α-Smooth Muscle Actin (α-SMA), anti-transmembrane protein 119 (TMEM119), anti-3-nitrotyrosine (3-NT) and anti-phosphorylated Tau (p-Tau) were purchased from Abcam (Cambridge, MA, USA). Anti-3-nitrotyrosine, anti-IL1β, anti-IL6, anti-brain-derived neurotrophic factor (BDNF), anti-CD40, and anti-β-actin primary antibodies were purchased from Santacruz Biotechnology (Dallas, TX, USA). Anti-insulin receptor substrate 1 (IRS1) primary antibody was purchased from Cell Signaling Technology (Danvers, MA, USA). Anti-glial fibrillary acidic protein (GFAP) and anti-S100β primary antibodies were purchased from Proteintech (Rosemont, IL, USA). Species-specific biotinylated secondary antibodies and streptavidin-HRP (Vectastain Elite ABC kit) were purchased from Vector Laboratories (Burlingame, CA, USA). Fluorescence-conjugated (Alexa Fluor) secondary antibodies and ProLong Diamond antifade mounting media with 4′,6-diamidino-2-phenylindole (DAPI) were purchased from Thermofisher Scientific (Grand Island, NY, USA). All other chemicals used in this study were purchased from Sigma unless otherwise specified. Animal tissues were sent to AML laboratories (Baltimore, MD, USA) and Instrument Resources Facility, University of South Carolina School of medicine (Columbia, SC, USA), for paraffin embedding and sectioning. Microbiome analysis was performed at Cosmos ID (Rockville, MD, USA).

### 2.2. Animals

C57BL/6J adult wild-type male mice (10 weeks) were purchased from the Jackson Laboratories (Bar Harbor, ME, United States) and used in this study. Mice were implemented in accordance with National Institutes of Health (NIH) NIH guidelines for human care and use of laboratory animals and local Institutional Animal Care and Use Committee (IACUC) standards. The animal handling procedures were approved by University of South Carolina, Columbia, SC, United States. Upon arrival, all mice were housed at 22–24 °C with a 12 h light/12 h dark cycle and ad libitum access of food and water. According to our experimental design, all mice were fed either with normal chow diet from Teklad (Madison, WI, USA) or Western diet from Research Diets (New Brunswick, NJ, USA). On completion of animal experiments, all mice were sacrificed. Organs including liver and distal part of small intestine were collected and fixed in 10% neutral buffered formaldehyde, whereas frontal cortex was collected and fixed in Bouin’s solution (Sigma Aldrich St. Louis, MO, USA). Preparation of serum samples was performed using fresh mice blood collected by cardiac puncture immediately after anesthesia of mice. Fecal pellets were obtained from the colon and preserved for microbiome analysis.

#### 2.2.1. Mouse Model of Gulf War Illness (GWI)

Mice were exposed to GW chemicals (PB + PER) as described in our previous studies [11,37]. After 1 week of acclimatization, all mice were randomly distributed into four treatment groups. The first and third groups of mice were given only vehicle (0.6% dimethyl sulfoxide (DMSO) ) for two weeks and denoted as CHOW (*n* = 6) and WD (*n* = 6), respectively. The second and fourth group of mice were treated with a combination of GW chemicals PB (2 mg/kg body weight and diluted in PBS) and Per (200 mg/kg body weight, diluted in DMSO and PBS) tri-weekly for two weeks by oral gavage and denoted as CHOW+GWI (*n* = 6) and WD+GWI (*n* = 6), respectively. All the mice groups were fed with chow diet during the initial two weeks of GW chemical exposure. Following GW chemical exposure for 2 weeks, both the WD and WD+GWI mice groups were shifted to Western diet continuously for 20 weeks, whereas both the CHOW and CHOW+GWI mice groups were continued with normal chow diet. The Western diet (Research Diets, Cat#12079B) used for this study has 17% kcal protein, 40% kcal fat, and 43% kcal carbohydrate distribution. Fecal pellets from these mice were sent for microbiome analysis. Blood, distal small intestine segments, liver tissue and frontal cortex tissue segments were collected following sacrifice 5 months post-GW chemical exposure. Enzyme-Linked Immunosorbent Assay (ELISA) was performed from serum of 4–5 mice samples per group.

#### 2.2.2. Microbiome Analysis

Fecal pellets from each experimental mouse were collected in a sterile environment and snap frozen in liquid nitrogen for microbiome analysis at CosmosID (Rockville, MD, USA). Briefly, total DNA isolation and purification were performed using the ZymoBIOMICS Miniprep kit. Library preparation and quantification were performed using Qubit dsDNA HS assay (ThermoFisher, Waltham, MA, USA) and qualified on a 2100 bioanalyzer instrument (Agilent, Santa Clara, CA, USA) to show a distribution with a peak in the expected range. Further, whole-genome shotgun sequencing (WGS) was performed using the next-generation sequencing (NGS) platform and according to the vendor-optimized protocol. The unassembled sequencing reads for each sample were analyzed by the CosmosID bioinformatics platform. The Diverging bar charts were generated using the phylum- and species-level relative abundance matrices from the taxonomic analysis. All values were converted to their Log2 distance from the organism’s average across all groups. Linear Discriminant Analysis Effect Size (LEfSe) figures were generated using the LefSe tool from the Huttenhower lab, based on phylum- and species-level relative abundance matrices from taxonomic analysis [38]. LefSe is calculated with a Kruskal–Wallis alpha value of 0.05, a Wilcoxon alpha value of 0.05, and a logarithmic Linear Discriminant Ananlysis (LDA) score threshold of 2.0. In the LefSe figures, red bars to the right convey that the organism in that group is more abundant in the “WD+GWI” group than the “CHOW+GWI” group. Green bars to the left convey that the organism is more abundant in the “CHOW+GWI” group. Stacked bar figures were generated from the phylum- and species-level relative abundance matrices from the taxonomic analysis. Stacked bar figures for each group were generated using the R package ggplot2. Heat maps were creating using the NMF R package, based on the relative abundance information from the taxonomic analysis. A hierarchically clustered heat map is provided. Alpha diversity boxplots were calculated from the species-level abundance score matrices from the taxonomic analysis. Chao, Simpson, and Shannon alpha diversity metrics were calculated in R using the R package Vegan. Beta Diversity Principal Coordinate Analyses were calculated from the species-level relative abundance matrices from the taxonomic analysis. Bray–Curtis diversity was calculated in R using the R package Vegan with the function vegdist. Beta diversity PCoA tables were generated using Vegan’s function PCoA. PERMANOVA tests for each distance matrix were generated using Vegan’s function Adonis2.

#### 2.2.3. Mouse Microglial Cell Culture and Treatment

Immortalized mouse microglial cells SIM-A9 were purchased from ATCC (ATCC^®^ CRL-3265), subcultured and grown on Dulbecco’s Modified Eagle Medium/F12 (DMEM/F12), 10% fetal bovine serum (FBS) and heat-inactivated 5% horse serum. The cells were incubated at 37 °C using a humidified CO_2_ incubator (5% CO_2_ concentration). 

Approximately 0.05 × 10^6^ number of cells were seeded onto a 24-well tissue culture plate and growth was permitted to reach 70% confluency before the experiment. Serum starvation was achieved using DMEM/F12 media, supplemented with 1% FBS for at least 18 h. Following sera starvation, cells were treated with serum (25 µL/mL) obtained from the CHOW, CHOW+GWI, WD, and WD+GWI mice groups, respectively, or PBS (VEH), or LPS (1 µg/mL) for 24 h. Cells were harvested for in vitro immunofluorescence experiments and supernatants were collected for measuring IL6 by enzyme-linked immunosorbent assay (ELISA) and cellular cytotoxicity assay.

#### 2.2.4. Immunohistochemistry

Deparaffinization of paraffin-embedded small intestine, liver, and brain tissue sections was performed following the standard laboratory procedure. Briefly, tissues were immersed successively in 100% xylene, 1:1 solution of xylene and ethanol, 100% ethanol, 95% ethanol, 70% ethanol, 50% ethanol, and deionized water for 3 min each. Following deparaffinization, antigen epitope retrieval was performed using the epitope retrieval solution and steamer (IHC-World, Woodstock, MD, USA). Endogenous peroxidase activity was blocked using 3% H_2_O_2_ solution for 20 min, followed by serum blocking (5% goat serum) for 1 h. After serum blocking, primary antibodies for IL1β, IL6, CD68, α-SMA, and BDNF were diluted (1:300) in blocking buffer and applied on the tissue sections. All sections were kept at 4 °C for overnight incubation in a humidified chamber. After overnight incubation, the tissue sections were washed with 1X PBS-T (PBS + 0.05% Tween 20) 3 times. Biotinylated secondary antibodies (species specific) were probed at 1:250 dilution, followed by incubation with streptavidin conjugated with horseradish peroxidase at 1:500 dilution. Finally, the chromogenic substrate solution of 3,3-diaminobenzidine (DAB) (Sigma-Aldrich) was applied on the sections and counterstaining was performed using Mayer’s hematoxylin (Sigma-Aldrich). Mounting of all tissue sections was performed using Simpo mount (GBI Laboratories, Mukilteo, WA, USA). Reactivity of the applied antibodies in the sections was observed under 10X and 20X objectives and images were captured using an Olympus BX43 microscope (Olympus, Center Valley, PA, USA). Morphometric data analyses were performed using CellSens Software from Olympus America (Center Valley, PA, USA). 

#### 2.2.5. Immunofluorescence Staining

Deparaffinization and epitope retrieval procedures of paraffin-embedded small intestine and brain tissue sections were performed as described previously. Following the epitope retrieval process, the tissue sections were permeabilized using PBS-T (PBS + 0.1% Triton X-100) solution for 1 h. Blocking was performed using 5% goat serum and the sections were incubated with primary antibodies of anti-Occludin, anti-Claudin-2, anti-GFAP, anti-S100β, anti-CD40, and anti-TMEM119 (at 1:300 dilution) and kept at 4 °C overnight. Anti-IgG secondary antibodies (species specific) conjugated with Alexa Fluor 488 or 633 (Invitrogen) were used at 1:250 dilutions. Tissue sections were mounted using ProLong Gold antifade reagent with DAPI (Life Technologies, Carlsbad, CA, USA). Images were taken under 40× (for in vivo experiments) and 60× magnifications (for in vitro experiments) with an Olympus BX63 microscope.

#### 2.2.6. Western Blot

Western blot was performed using the proteins extracted from liver and frontal cortex of mice tissues. Protein samples were extracted using RIPA lysis buffer containing protease and phosphatase inhibitors, followed by estimation of the extracted tissue proteins by the BCA assay kit (Thermo Fisher Scientific, Rockford, IL, USA). Approximately 30 µg of proteins extracted from each sample, were added to a mixture containing 1X NuPAGE™ LDS Sample Buffer (Thermo Fisher Scientific, Rockford, IL, USA) and 10% β-mercaptoethanol, and then boiled for 5 min for denaturation purposes. The protein samples were subjected to standard SDS-PAGE using Novex 4–12% bis-tris gradient gel and nitrocellulose membrane transfer of resolved protein bands was performed using the Trans-Blot Turbo transfer system (Bio-rad, Hercules, CA, USA). Following Ponceau S staining, membrane blocking was performed with 5% bovine serum albumin (BSA) for 1 h. Primary antibodies including anti-IRS-1, anti-p-Tau, and anti-β-actin were diluted (1:1000) and probed overnight at 4 °C. Compatible horseradish peroxidase-conjugated species-specific secondary antibodies were used to tag the primary antibody. For the development of the blot, Pierce ECL Western blotting substrate (Thermo Fisher Scientific, Waltham, MA, USA) was used. Finally, the blots were captured by G: Box Chemi XX6 and densitometry analysis was performed using Image J software.

#### 2.2.7. Serum ELISA

Serum collected from the CHOW, CHOW+GWI, WD, and WD+GWI mouse groups was used to quantify IL6 and insulin concentration using commercially available ELISA kits from Thermo Fisher Scientific (Waltham, MA, USA) and Crystal Chem (Elk Grove Village, IL, USA). IL6 ELISA was also performed using supernatant collected from mouse microglial SIM-A9 cells. The ELISA procedures were performed according to the manufacturer’s protocol.

#### 2.2.8. Cell Cytotoxicity Assay

Cell cytotoxicity was determined using 100 μL of supernatant from the sera-treated SIM A9 cells by the LDH-Cytotoxicity Colorimetric Assay Kit (BioVision, Milpitas, CA, USA) following the manufacturer’s protocol. The cytotoxicity was calculated using the formula (supplied with the kit): Cytotoxicity (%) = (Test Sample − Low Control)/(High Control − Low Control) × 100. Low control was cells in DMEM/F12 media with 1% serum and high control was cells in DMEM/F12 media with 1% serum along with 1% Triton X-100.

### 2.3. Statistical Analyses

All experiments were performed with 5–6 mice per group and each individual mouse was treated as a single sample. The statistical analysis was carried out by unpaired t-test (two-tailed tests with equal variance) and one-way analysis of variance (ANOVA) for assessing the difference between multiple groups. For all analyses, *p* ≤ 0.05 was considered statistically significant, and data were presented as the mean ± SEM. Statistical significance was measured by Bonferroni–Dunn post-hoc analysis for all intergroup comparisons.

## 3. Results

### 3.1. Shotgun Metagenomics Show Alteration of Species Composition and Abundance in Western Diet-Fed Mice with GWI and Obesity

**Figure 1:**
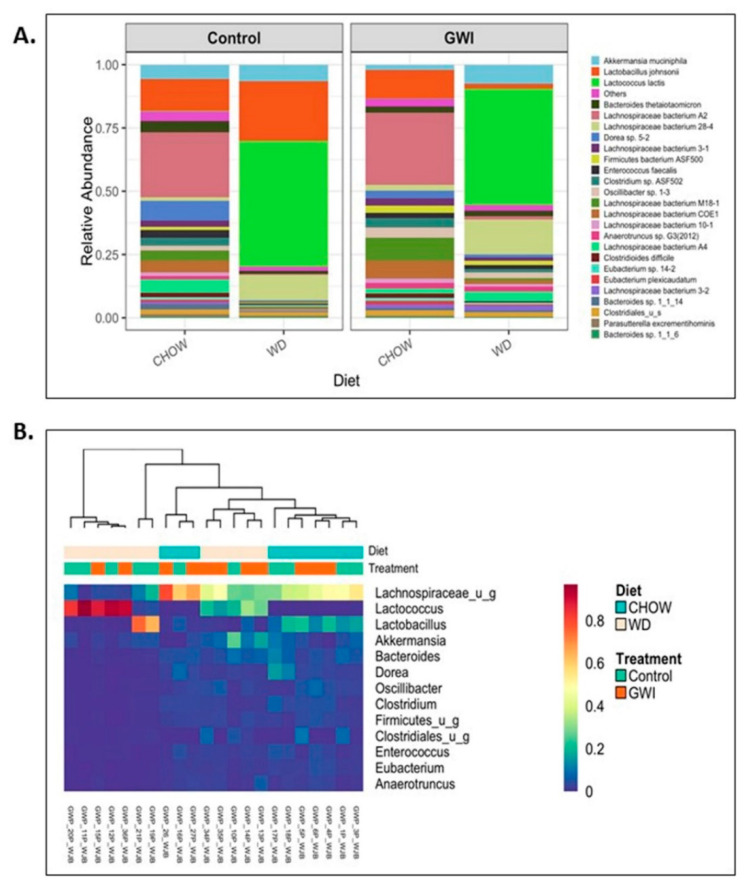
Obesity induces microbial dysbiosis in Gulf War illness (GWI) persistence. (**A**). A stacked bar graph presented the relative abundance of species in both the control and GWI groups. The left box (control) includes both the lean (CHOW) and obesity (WD) control groups. However, the right box (GWI) includes both GWI persistence in the lean mice (CHOW+GWI) and in the obese mice (WD+GWI) groups. (**B**). Relative abundance heatmap and hierarchical clustering at species levels in all study groups.

**Figure 2:**
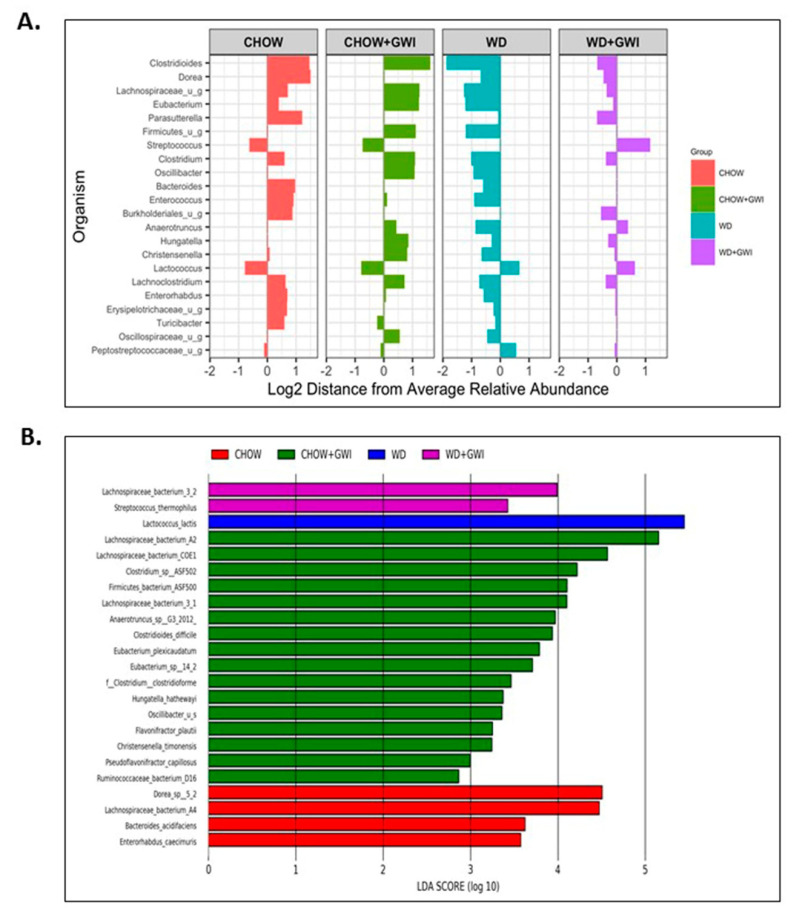

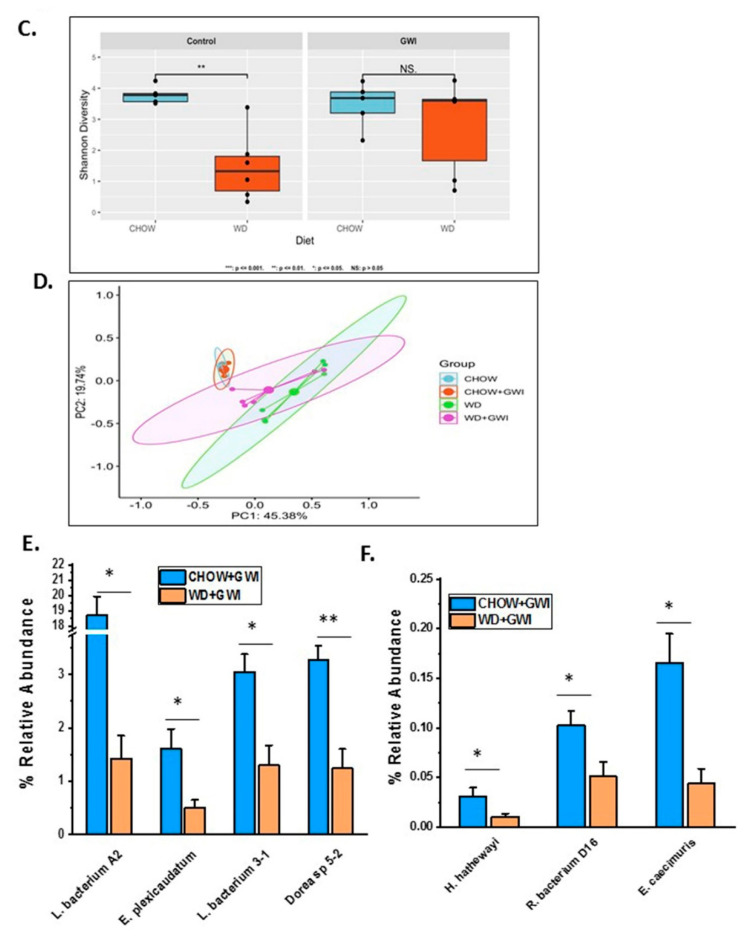
Obesity induces microbial dysbiosis at the species level in Gulf War illness (GWI) persistence. (**A**). Diverging bar (centroid) graph showing organisms’ log2 relative abundance at species levels in the CHOW (lean), CHOW+GWI (persistence Gulf War illness), WD (obese) and WD+GWI (persistence Gulf War illness underlying obesity) groups. (**B**). Differential species abundance (LEfSe analysis) among all study groups (CHOW, CHOW+GWI, WD and WD+GWI) is calculated using three methods: the Kruskal–Wallis sum-rank test, the Wilcoxin rank-sum test, and Linear Discriminant Analysis (LDA). *p* ≤ 0.01 for Kruskal–Wallis and Wilcoxon tests; LDA score ≥ 2.0 or ≤ −2.0. (**C**). Box plot showing α diversity (Shannon) in all experimental groups. The left box (control) includes both lean (CHOW) and obesity (WD) control groups. However, the right box (GWI) includes both GWI persistence in the lean mice (CHOW+GWI) and in the obese mice (WD+GWI) groups. (**D**). Bray–Curtis β diversity plot with 95% confidence ellipse in in all experimental groups including both the lean (CHOW) and obesity (WD) control groups, and both GWI persistence in the lean mice (CHOW+GWI) and in the obese mice (WD+GWI) groups. (**E**,**F**). Bar graphs showing the percent relative abundance of beneficial bacterial species. Data were presented as the mean of percent relative abundance with SEM. *t*-test analyses were performed. * denotes a significant level at *p* < 0.05 and ** denotes a significant level at *p* < 0.001, NS = not significant.

Shotgun metagenomic analysis of fecal contents was performed using next-generation sequencing to discern whether Western diet-induced obesity caused an altered species profile in the host of GWI mice. Results showed a marked observed difference in the species profiles and relative abundance among the Chow, Chow+GWI, WD, and WD+GWI groups (Figure 1A). Species diversity, richness, and abundance were also studied. Results show that several species and clusters were differentially altered between groups (Figure 1B). Detailed analysis of species diversity, richness, and abundance (via LEfSe analysis) showed that at *p* = 0.01, the species *Lactococcus lactis* and *Streptococcus thermophilus* were significantly increased in the WD+GWI group when compared to the Chow+GWI group (Figure 2B). However, at *p* = 0.05, the species *Akkermansia muciniphila* and *Staphylococcus xylosus* were also significantly increased in the WD+GWI group when compared to the CHOW+GWI group (Supplementary Figure S1). Sixteen bacterial species, including *Lachnospiraceae species*, *Clostridium*, and *Enterorhabdus*, dominated the abundance in the Chow+GWI group over and above the WD+GWI group (Figure 2A). Results also showed that the relative abundance of *Lactococcus lactis*, *Akkermansia muciniphila*, and *Lachnospiraceae bacterium 28-4* showed an observed difference in the WD+GWI group over and above the Chow+GWI group (Supplementary Figure S1). Shannon diversity analysis, which is used to describe species evenness and richness using abundance scores, showed a marked difference in the WD+GWI group when compared to the Chow+GWI group though the results were not significant at the *p* < 0.05 level. Interestingly, Shannon diversity indices were significantly different between the Chow and WD groups (Figure 2C). Beta diversity shows the difference in microbial species-level composition in an environment or treatment group and can be represented by Bray–Curtis distance. PERMANOVA testing showed that the Bray–Curtis beta diversity of WD+GWI compared to the Chow+GWI group was significantly different (Figure 2D). To show whether bacterial species that are known to exert a beneficial association to gut and neuronal health are present, analyses of the most abundant, seven such species were performed (Figure 2E,F). Results showed that butyrogenic bacteria, bacteria which contributes to good immune health, such as *Lachnospiraceae Bacterium A2*, *COE-1*, *3-1*, *10-1*, *Eubacterium species*, *Ruminococcacae*, *Enterohabdus* sp, and *Hungatella*, decreased significantly in the WD+GWI group when compared to the Chow+GWI group. However, *Dorea* sp, a bacterial species known to be associated with bloating, constipation and bad gut health, was significantly decreased in the WD+GWI group when compared to the Chow+GWI group (Figure 2E). The direct species comparison of individual bacteria among these two groups suggested strongly that a prolonged Western diet feeding and the resultant underlying obesity decrease bacterial species that are known to improve gut, neuronal and immune health [39,40,41,42] (Figure 2E,F).

### 3.2. Western Diet-Fed Mice with GWI Show Increased Serum Levels of Proinflammatory Cytokine IL6 and Hyperinsulinemia

**Figure 3:**
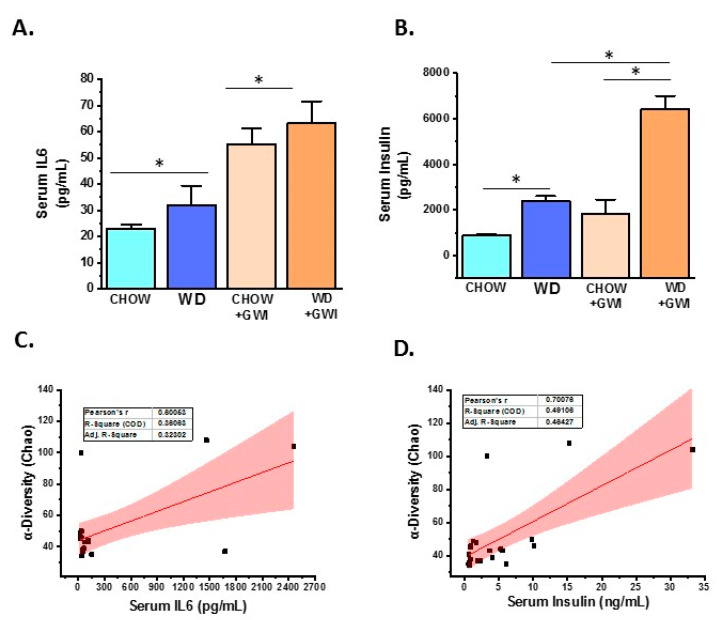
Obesity-induced microbial dysbiosis is associated with systemic IL6 levels and insulin resistance Gulf War illness (GWI) persistence. (**A**). Bar graph showing serum IL6 level (pg/mL) in all the CHOW (lean), CHOW+GWI (persistence Gulf War illness), WD (obese) and WD+GWI (persistence Gulf War illness underlying obesity) groups, and (**B**). Serum insulin level (pg/mL) in all groups—CHOW, WD, CHOW+GWI, and WD+GWI. Data in A and B were presented as the mean and SEM. *t*-test analyses were performed. * denotes a significant level at *p* < 0.05. (**C**,**D**). Correlation plot of microbiome α diversity index (chao) by serum IL6 (C), and by serum insulin (D). Results of multivariate analyses for association with serum ELISA measurements are shown. Pearson’s linear regression is shown in red with 95% confidence bands. IL6, Interleukin 6.

To show whether WD feeding and underlying obesity worsened serum levels of IL6, a reliable indicator of GWI pathology, serum ELISA was performed in mice. Results showed that IL6 levels were significantly increased in the WD+GWI group compared with the Chow+GWI group (Figure 3A). Notably, alpha diversity representing species abundance was strongly correlated (Pearson’s R) with increased IL6 levels in the blood of the WD+GWI group when compared to the chow+GWI group, suggesting a probable role of the microbiome diversity observed in WD+GWI mice in elevated serum IL6 (Figure 3C) (r = 0.6). Aging, an underlying obesity phenotype, and chronicity of GWI pathology, can often lead to increased insulin levels in the blood, signifying underlying metabolic disease. Insulin levels in WD+GWI mice showed a significant increase in its levels when compared to the Chow+GWI group (Figure 3B), and microbiome dysbiosis represented by alpha diversity was strongly associated with the insulin level increase in the blood of the WD+GWI group (Figure 3D). The results suggested that proinflammatory levels of IL6 and hyperinsulinemia were higher in underlying obesity with microbial dysbiosis represented by alpha diversity and might be a probable cause of such pathology.

### 3.3. Western Diet-Fed Mice with GWI Show Altered Tight Junction Protein Expression and Enteric Glial Cell Activation, Thus Worsening GWI Intestinal Pathology

**Figure 4:**
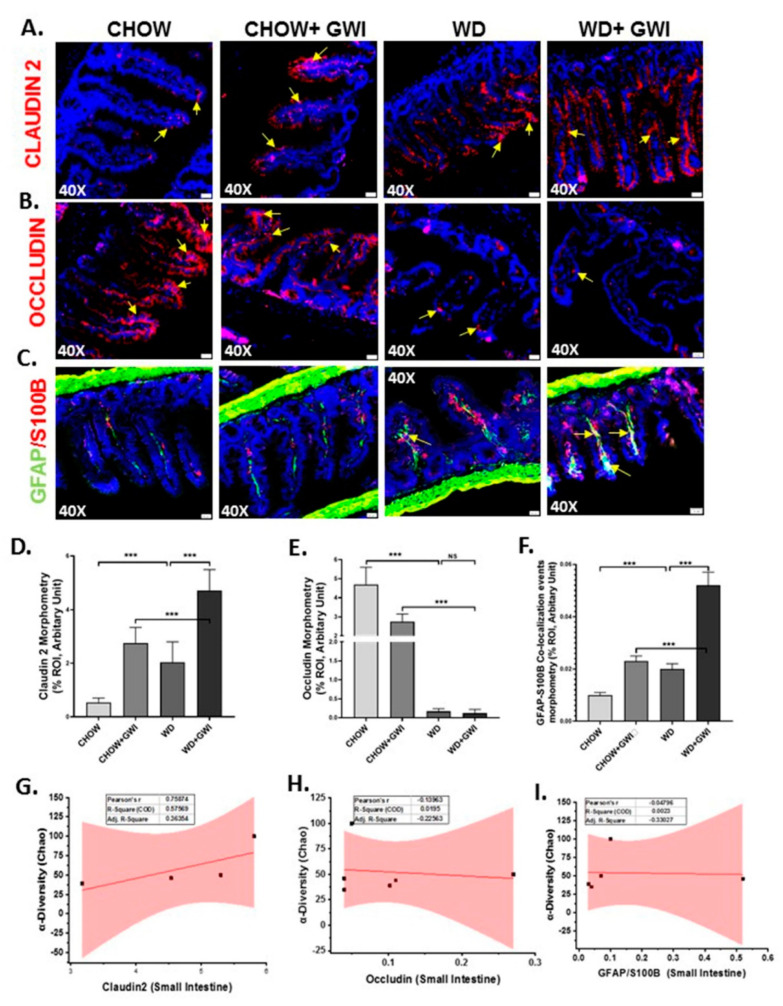
Tight junction protein levels, enteric glial cell activation, and their correlation with microbial species diversity in Gulf War Illness (GWI) following an obesogenic diet feeding. (**A**–**C**). Immunoreactivity of Claudin 2 (red) in (**A**), Occludin (red) in (**B**) and co-localization of GFAP/S100B (yellow) in (**C**) as shown by immunofluorescence microscopy in intestinal tissue sections from CHOW (lean), CHOW+GWI (persistence Gulf War illness), WD (obese) and WD+GWI (persistence Gulf War illness in underlying obesity) mice. Immunoreactivity of Claudin 2, Occludin, and co-localization of GFAP/S100B was indicated as yellow arrows. All images were taken at 40× magnification (50 µm). (**D**–**F**). Bar graphs showing immunoreactivity of Claudin 2 in (**D**), Occludin in (**E**) and GFAP/S100B co-localization (**F**). Results were displayed as the mean ± SD of ROI% of three different areas (*n* = 6). Significance was tested by unpaired *t*-test; *** *p* < 0.001, NS = non-significant. (**G**–**I**). Correlation plot of α diversity (Chao) of bacterial species index by immunoreactivity obtained from immunofluorescence of Claudin 2 as (**G**), Occludin as (**H**), and co-localized GFAP/S100B as (**I**) of the WD+GWI mouse group. Pearson’s linear regression is shown in red with 95% confidence bands. GFAP, Glial fibrillary acid protein; S100B, S100 calcium binding protein B.

We determined whether underlying obesity caused an alteration of tight junction proteins in the distant ileum by Claudin-2 and Occludin immunofluorescence analysis. Results showed that GWI mice that were fed with the Western diet showed significantly increased levels of Claudin-2 when compared to chow-fed mice (Chow+GWI) (Figure 4A,D) (*p* < 0.01). The WD+GWI group also showed significantly decreased levels of Occludin when compared to the Chow+GWI group (Figure 4B,E) (*p* < 0.01). Activation of enteric glial cells has been shown to be aiding in the proinflammatory events in the intestine and contribute to GWI intestinal pathology [12]. Activation of these cells, as shown by the co-localization of GFAP/S100B, was significantly increased in the WD+GWI group when compared to the Chow+GWI group (Figure 4C,F) (*p* < 0.01). The co-localization events are shown by yellow pointed arrows. To show whether the microbial diversity was associated with the intestinal changes in pathology, a correlation analysis was carried out. Results showed that microbial diversity was strongly correlated with an increase in Claudin 2 levels in the WD+GWI group (r = 0.7) (Figure 4G). However, both Occludin levels and activation of enteric glial cells were not correlated with microbial species diversity and abundance in the WD+GWI group (Figure 4H,I). The results suggested that the underlying obesity following Western diet feeding was responsible for altered expression of tight junction protein, especially Claudin-2 and Occludin and concomitant activation of enteric glial cells, but microbiome diversity was only able to influence Claudin-2 protein levels in the obesity phenotype.

### 3.4. Western Diet-Fed Mice with GWI Show Altered Tissue Proinflammatory Mediators IL1β and IL6, Thus Worsening GWI Intestinal Pathology

**Figure 5:**
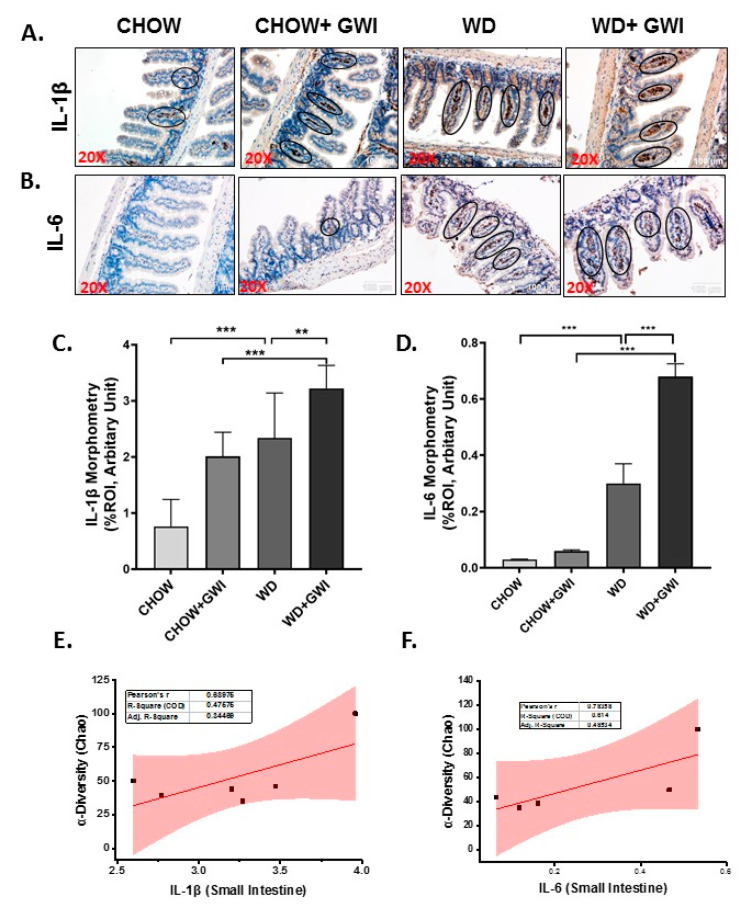
Gastrointestinal inflammation and its correlation with microbial species diversity in Gulf War illness (GWI) following obesogenic diet feeding. (**A**,**B**). Immunoreactivity of inflammatory markers IL1β (**A**) and IL6 (**B**) proteins was shown by immunohistochemistry in intestinal tissue sections from the CHOW (lean), CHOW+GWI (persistence Gulf War illness), WD (obese) and WD+GWI (persistence Gulf War illness underlying obesity) mice groups. Immunoreactivity was indicated by black circles. Images were taken at 20× magnification (scale 100 µm). (**C**,**D**). Bar graph showing immunoreactivity of IL1β as (**C**), and IL6 as (**D**). Results were displayed as the mean ± SD of ROI% of 3 different areas (*n* = 6). Significance was tested by unpaired *t*-test; ** *p* < 0.01, and *** *p* < 0.001. (**E**,**F**). Correlation plot of α diversity (Chao) of bacterial species index by immunoreactivity obtained from immunohistochemistry of IL1β as (**E**) and IL6 as (**F**) of the WD+GWI mouse group. Pearson’s linear regression is shown in red with 95% confidence bands. IL1β, Interleukin 1 beta; IL6, Interleukin 6.

To show whether the underlying obesity worsens the release of proinflammatory cytokines from the intestinal segments, immunoreactivities of IL1β and IL6 were studied using immunohistochemistry. Results showed that IL1β tissue levels were increased significantly in the WD+GWI group when compared to the Chow + GWI group (Figure 5A,C) (*p* < 0.01), while IL6 tissue levels also followed a similar pattern when compared similarly (Figure 5B,D) (*p* < 0.01). Notably, both IL1β and IL6 tissue levels were positively correlated with microbiome diversity in the gut (Figure 5E,F) (r = 0.68; 0.78). The results suggested that the underlying obesity following Western diet feeding worsened the GWI intestinal inflammation, as shown by increased levels of IL1β and IL6. Further, the microbiome diversity evident from abundance scores positively correlated with the increased levels of the above proinflammatory mediators. 

### 3.5. Western Diet-Fed Mice with GWI Show Altered Hepatic Kupffer Cell Activation and Profibrotic Phenotype, Thus Worsening GWI Hepatic Pathology

**Figure 6:**
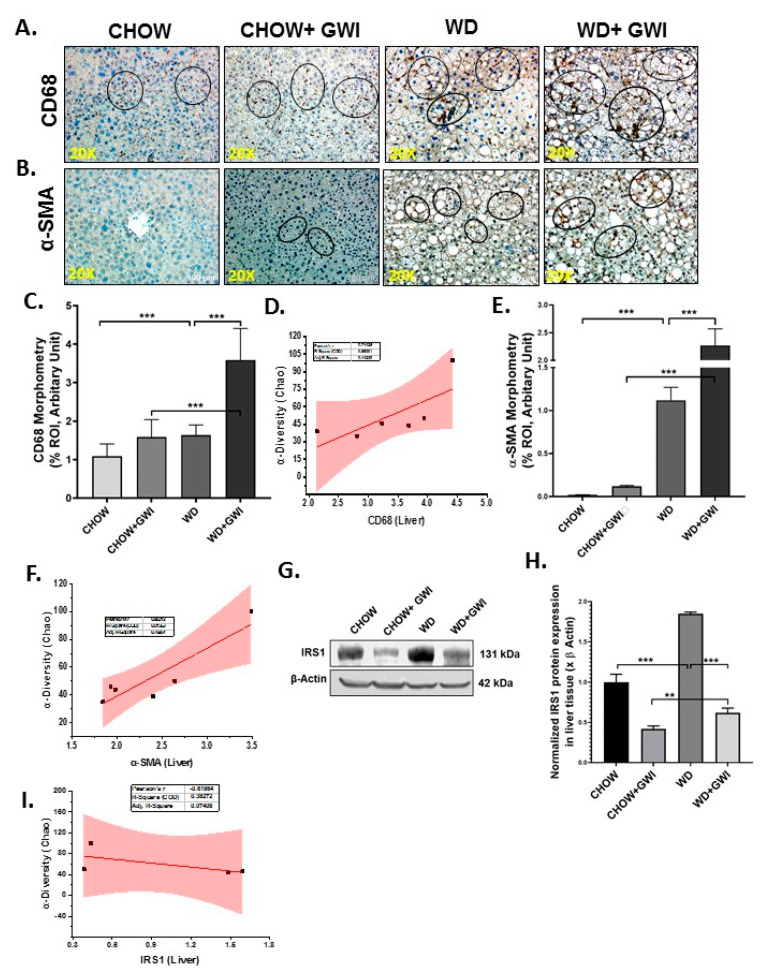
Kupffer cell and stellate cell activation in the liver and their correlation with microbial species diversity in Gulf War Illness (GWI) following obesogenic diet feeding. (**A**,**B**). Immunoreactivity Kupffer cell activation marker CD 68 (**A**) and stellate cell activation marker α-SMA (**B**) proteins were shown by immunohistochemistry in liver sections from the CHOW (lean), CHOW+GWI (persistence Gulf War illness), WD (obese) and WD+GWI (persistence Gulf War illness underlying obesity) mice groups. Immunoreactivity was indicated by black circles. Images were taken at 20× magnification (scale 100 µm). (**C**,**E**). Bar graph showing immunoreactivity of CD 68 as (**C**), and α-SMA as (**E**). Results were displayed as the mean ± SD of ROI% of three different areas (*n* = 6). Significance was tested by unpaired *t*-test; ** *p* < 0.01, and *** *p* < 0.001. (**G**). Immunoblot analyses of IRS-1 from liver tissue lysates of the CHOW, CHOW+GWI, WD, and WD+GWI mouse groups. (**H**). Densitometric analyses of IRS-1 immunoreactivity displayed as the mean ± SD (*n* = 3), normalized against β-actin, and plotted as a bar graph. (**D**,**F**,**I**). Correlation plot of α diversity (Chao) of bacterial species index by immunoreactivity obtained from immunohistochemistry of CD 68 as (**D**) and α-SMA as (**F**) and immunoblot of IRS-1 (**I**) of the WD+GWI mouse group. Pearson’s linear regression is shown in red with 95% confidence bands. CD68, Cluster of Differentiation 68; α-SMA, alpha-smooth muscle actin; IRS1, insulin receptor substrate 1.

GWI patients hardly report liver abnormalities in the clinics owing to the silent nature of liver pathology especially related to fatty liver or its most progressive inflammatory form though obstructive liver disease cases have been documented [43,44]. We have shown previously that underlying liver inflammation is prevalent in mice models with the absence of any liver inflammatory foci and fibrotic pathology, a situation similar to early non-alcoholic fatty liver disease [44]. We determined whether a Western diet feeding would worsen liver pathology in GWI mice by examining CD68 (a Kupffer cell activation marker), and a-SMA (a myofibroblast phenotype marker) immunoreactivity. Results showed that CD68 levels were significantly increased in the livers of the WD+GWI group when compared to the Chow+GWI group (Figure 6A,C) (*p* < 0.01). Results also showed that α-SMA levels were significantly increased in the livers of the WD+GWI group when compared to the Chow+GWI group (Figure 6B,E) (*p* < 0.01). Notably, hepatic levels of insulin receptor substrate 1 (IRS-1), an important mediator in insulin resistance, were significantly decreased in the WD+GWI group when compared to the Chow+GWI group, suggesting that Western diet feeding worsened insulin resistance (Figure 6G,H). The data for IRS-1 also corroborated the increased hyperinsulinemia in the WD+GWI group observed earlier. The increased diversity of the microbial species in the WD+GWI group correlated positively with increased CD68 and α-SMA (Figure 6D,F) (r = 0.74 and 0.9, respectively), suggesting that increased hepatic inflammation and stellate cell activation for a probable profibrotic phenotype is strongly associated with increased species diversity. However, there was a negative correlation between increased species diversity and IRS-1 protein levels, suggesting that microbiome changes may be aiding in the observed insulin resistance (Figure 6I) (r= −0.6).

### 3.6. Western Diet-Fed Mice with GWI Show Heightened Neuroimmune Activation, Thus Worsening GWI Neuronal Pathology

**Figure 7:**
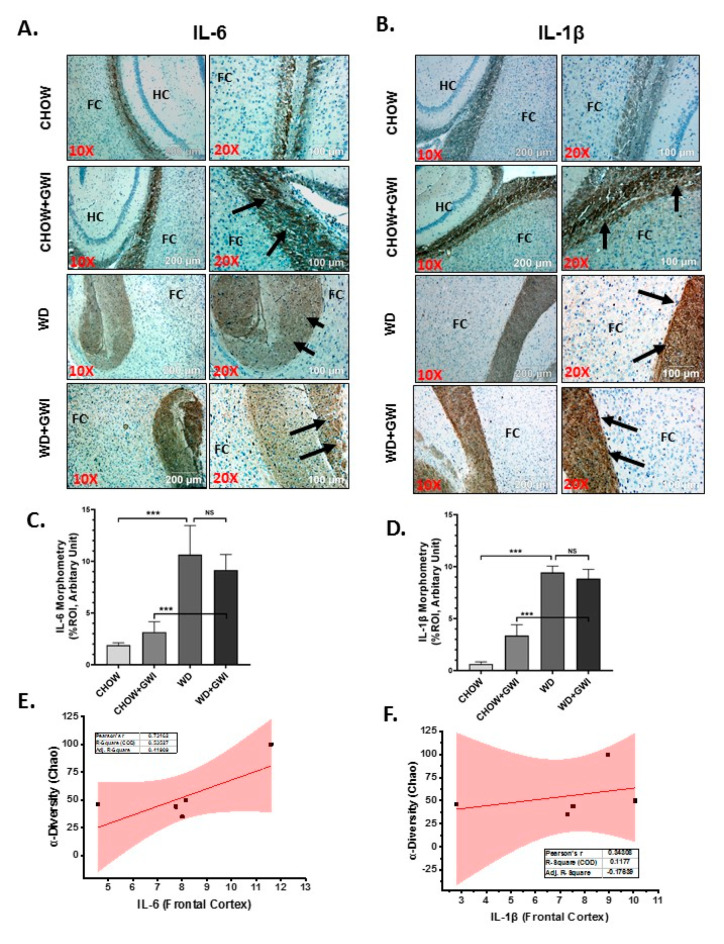
Neuroinflammation and its correlation with microbial species diversity in Gulf War Illness (GWI) following obesogenic diet feeding. (**A**) and (**B**). Immunoreactivity of neuroinflammatory markers IL6 (**A**) and IL1β (**B**) proteins was shown by immunohistochemistry in brain slices (the focused area is cerebral cortex) from the CHOW (lean), CHOW+GWI (persistence Gulf War illness), WD (obese) and WD+GWI (persistence Gulf War Illness underlying obesity) mice groups. Immunoreactivity was indicated by black arrows. Images were taken at 10× and 20× magnification. The frontal cortex is marked as FC, and HC is the hippocampus. (**C**,**D**). Bar graph showing immunoreactivity of IL6 as (**C**), and IL1β as (**D**). Results were displayed as the mean ± SD of ROI% of three different areas (*n* = 6). Significance was tested by unpaired *t*-test; *** *p* < 0.001; NS = non-significant. (**E**,**F**). Correlation plot of α diversity (Chao) of bacterial species index by immunoreactivity obtained from immunohistochemistry of IL6 as (**E**) and IL1β as (**F**) of the WD+GWI mouse group. Pearson’s linear regression is shown in red with 95% confidence bands. IL1β, Interleukin 1 beta; IL6, Interleukin 6.

**Figure 8:**
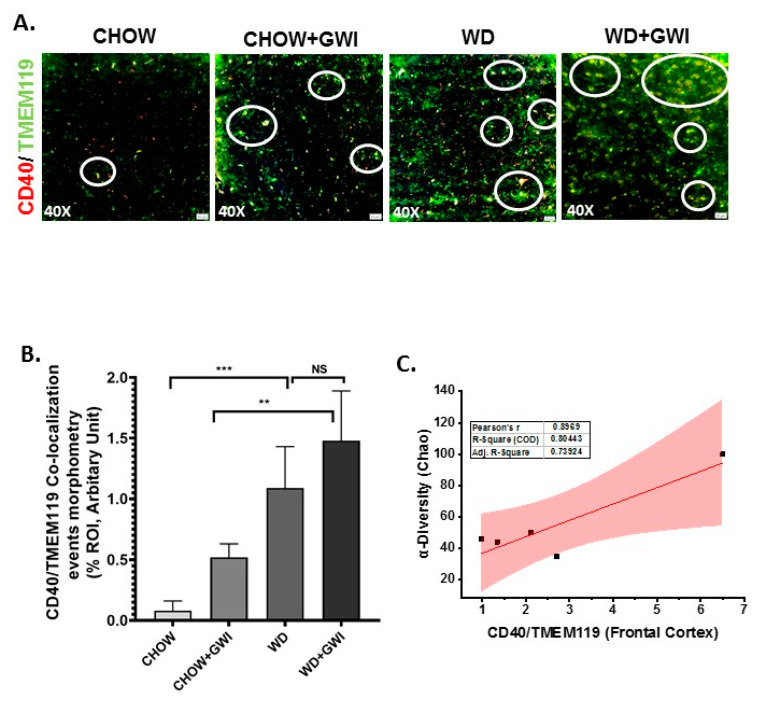
Microglial activation in the frontal cortex and its correlation with microbial species diversity in Gulf War illness (GWI) following an obesogenic diet feeding. (**A**). Co-localization of TMEM119/CD40 (yellow), as shown by immunofluorescence microscopy in frontal cortex sections from the CHOW (lean), CHOW+GWI (persistence Gulf War illness), WD (obese) and WD+GWI (persistence Gulf War illness underlying obesity) mice groups. Immunoreactivity was indicated by white circles. All images were taken at 40× magnification (50 µm). (**B**). Bar graph showing immunoreactivity of TMEM119/CD40 co-localization. Results were displayed as the mean ± SD of ROI% of three different areas (*n* = 6). Significance was tested by unpaired *t*-test; ** *p* < 0.01, and *** *p* < 0.001; NS = non-significant. (**C**). Correlation plot of α diversity (Chao) of bacterial species index by immunoreactivity obtained from immunofluorescence of co-localized TMEM119/CD40 of the WD+GWI mouse group. Pearson’s linear regression is shown in red with 95% confidence bands. CD40, Cluster of Differentiation 40; TMEM119, Transmembrane protein 119.

Gulf War illness is perceived as a neuroimmune disease [22,23,24,25]. Chemical exposures during the war theater have been considered as significant effectors of neuronal pathology. Further, the neuronal pathology persists even 28 years after the war ended. We have shown previously that microbial dysbiosis connects neuroinflammation via mediators of the persistent dysbiosis. We ascertained whether the underlying obesity due to Western diet feeding would worsen persistence of neuroinflammation and related pathology through immunohistochemistry for proinflammatory mediators in the frontal cortex. Results showed that IL6 and IL1β tissue levels in the frontal cortex increased significantly in the WD+GWI group when compared to the Chow+GWI group (Figure 7A–D) (*p* < 0.01). Microglia, the brain-specific macrophages with an active role in neuroinflammation, have been shown to play a significant role in GWI pathology. Results showed that levels of CD40 co-localization events with microglial marker TMEM119 were significantly increased in the WD+GWI group when compared to the Chow+GWI group (Figure 8A,B) (*p* < 0.01). To ascertain whether the dysbiosis shown earlier at the species level correlated with the observed neuroinflammation both for the proinflammatory cytokines and microglial activation, a Pearson’s R was calculated for each of these comparison parameters. Results showed that levels of frontal cortex IL6 and IL1β were correlated with alpha diversity of the microbiome species in the same group (WD+GWI) (Figure 7E,F) (r = 0.73 for IL6) while the same positive correlation was observed for microglial activation (Figure 8C) (r = 0.89). The results suggested that obesity worsened neuronal inflammation and microglial activation, and the microbial species diversity was strongly associated with the brain inflammatory pathology.

### 3.7. Western Diet-Fed Mice with GWI Show Decreased Tissue Specific Content of Neurotrophic Factor BDNF and a Concomitant Rise in Tau Phosphorylation, Thus Worsening the Risk of GWI Neurocognitive Deficiencies

**Figure 9:**
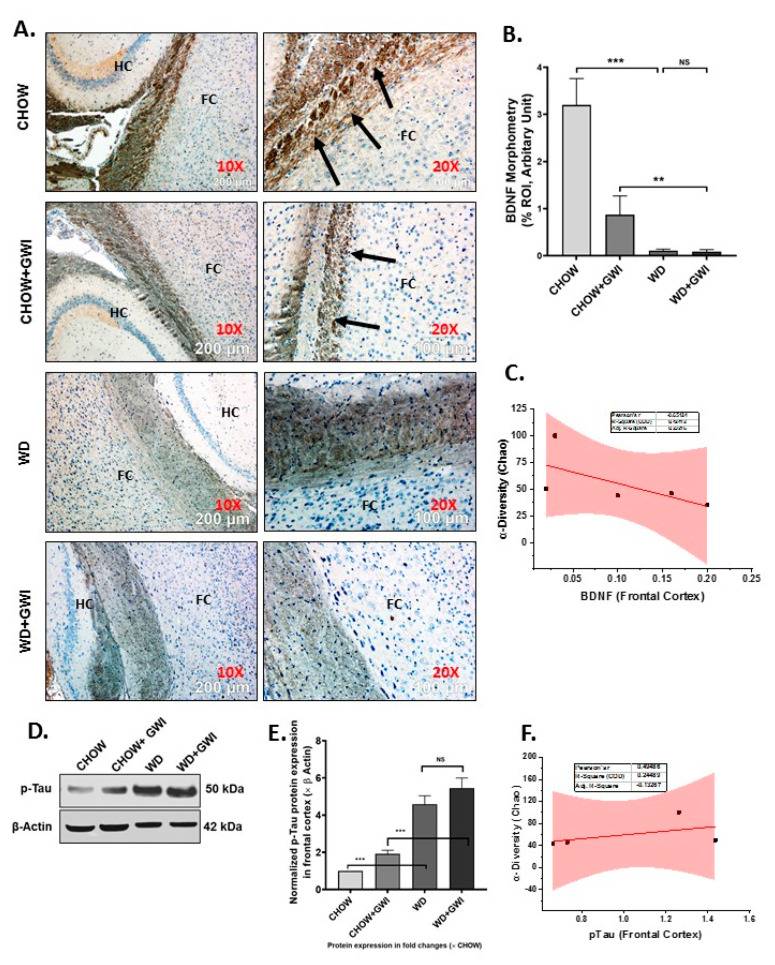
BDNF and accumulation of p-Tau and its correlation with microbial species diversity in Gulf War Illness (GWI) following an obesogenic diet feeding. (**A**). Immunoreactivity of neurotrophic marker BDNF protein was shown by immunohistochemistry in brain slices (the focused area is cerebral cortex) from the CHOW (lean), CHOW+GWI (persistence Gulf War illness), WD (obese) and WD+GWI (persistence Gulf War illness underlying obesity) mice groups. Immunoreactivity was indicated by black arrows. Images were taken at 10× and 20× magnification. The frontal cortex is marked as FC and the hippocampus as HC. (**B**). Bar graph showing immunoreactivity of BDNF. Results were displayed as the mean ± SD of ROI% of three different areas (*n* = 6). Significance was tested by unpaired *t*-test; ** *p* < 0.01, and *** *p* < 0.001; NS = non-significant. (**D**). Immunoblot analyses of p-Tau from frontal cortex tissue lysates of the CHOW, CHOW+GWI, WD, and WD+GWI mouse groups. (**E**). Densitometric analyses of p-Tau immunoreactivity displayed as the mean ± SD (*n* = 3), normalized against β-actin and plotted as a bar graph. (**C**,**F**). Correlation plot of α diversity (Chao) of bacterial species index by immunoreactivity obtained from immunohistochemistry of BDNF (**C**) and immunoblot of p-Tau (**F**) of the WD+GWI mouse group. Pearson’s linear regression is shown in red with 95% confidence bands. BDNF, Brain-derived neurotrophic factor; p-Tau, phosphorylated Tau.

GWI symptom persistence in patients often reflects cognitive and memory deficits. Notably, neuroinflammation and microglial activation have been shown to cause decreased BDNF release, which has a notable role in neurogenesis and synaptic plasticity. Further, the accumulation of phosphorylated Tau protein exhibits a strong correlation with memory and recognition problems. We uncovered whether the underlying obesity decreased the tissue content of BDNF and increased phosphorylated Tau in the frontal cortex by immunohistochemistry on brain slices. Results showed that BDNF levels were significantly decreased in the frontal cortex of the WD+GWI group when compared to the Chow+GWI group (Figure 9A,B) (*p* < 0.05). Phosphorylated Tau assessment by immunoblot showed a significant increase in the protein levels in the frontal cortex of the WD+GWI group when compared to the Chow+GWI group (Figure 9D,E) (*p* < 0.05). Notably, microbial species diversity as assessed by alpha diversity was negatively correlated with levels of BDNF (Figure 9C) (r = 0.65) and positively correlated with levels of phosphorylated Tau protein (Figure 9F, r = 0.6). The results above suggested that the underlying obesity due to Western diet feeding for a prolonged time decreases neurotrophic mediator BDNF while increasing accumulation of phosphor Tau protein, a mediator for defects in memory and Alzheimer-like pathology.

### 3.8. Mouse Serum from Mice with GWI and Underlying Obesity with an Altered Microbiome Activate Microglial Cells, Cause M1 Polarization and Induce Oxidative Stress and Cytotoxicity

**Figure 10:**
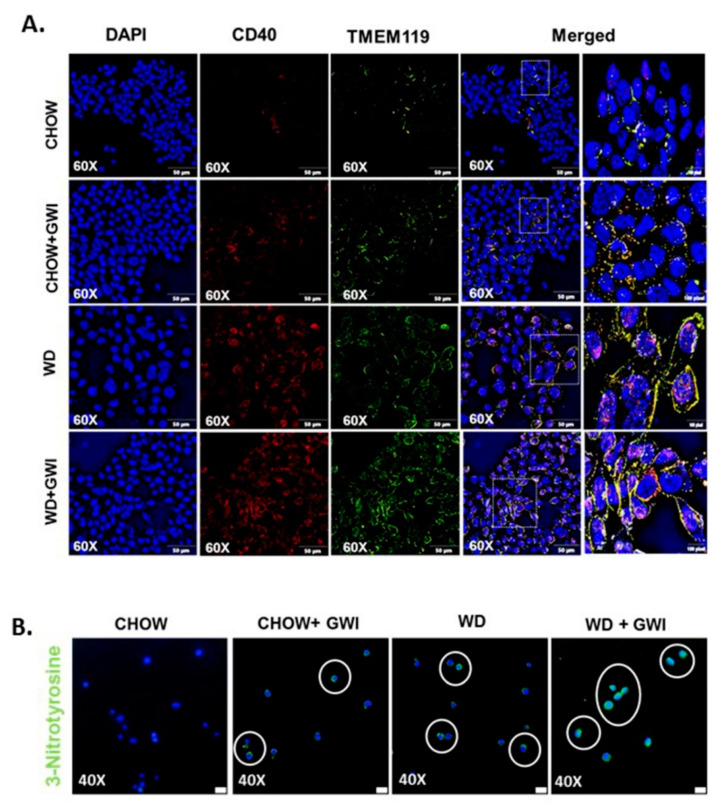

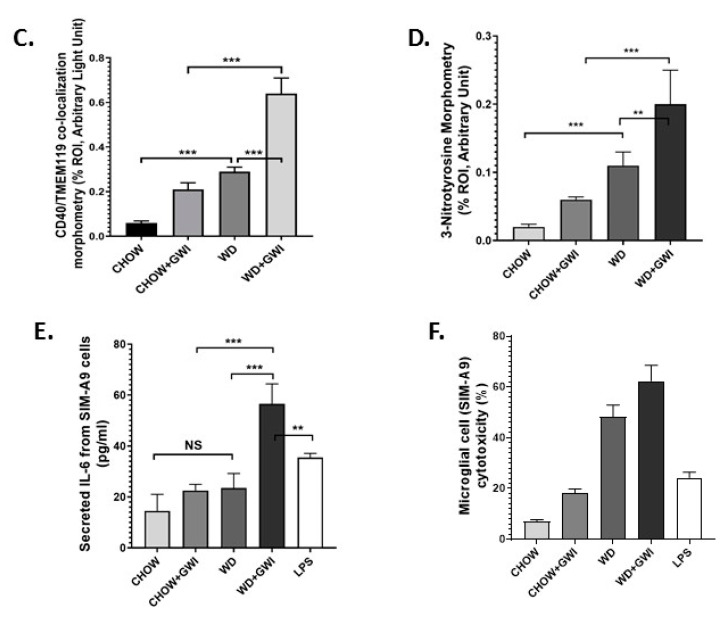
Obese mice serum induces microglial activation, tyrosine nitration, and IL6 release in mouse SIM-A9 cells. (**A**). Co-localization of CD40/TMEM119 (yellow) as shown by immunofluorescence microscopy in mouse microglial cells SIM-A9 treated with serum from CHOW (lean), CHOW+GWI (persistence Gulf War illness), WD (obese), and WD+GWI (persistence Gulf War illness underlying obesity). Nucleus was stained with DAPI. Images were taken at 60× magnification and a magnified image of the merged channels is presented at the right side. (**B**). Immunoreactivity of 3-nitrotyrosine as shown by immunofluorescence microscopy in mouse microglial cells SIM-A9 treated with serum from CHOW, CHOW+GWI, WD, and WD+GWI. Immunoreactivity was indicated as white circles. Nucleus was stained with DAPI. Images were taken at 40× magnification. (**C**,**D**). Bar graph showing immunoreactivity of CD40/TMEM119 co-localization (**C**) and 3-nitrotyrosin (**D**). Results were displayed as the mean ± SD of ROI% of three different areas (*n* = 3). Significance was tested by unpaired *t*-test; ** *p* < 0.01, and *** *p* < 0.001; NS = non-significant. (**E**). IL6 concentration (pg/mL) in supernatants from SIM-A9 cells treated with serum from CHOW, CHOW+GWI, WD, WD+GWI mice groups and with lipopolysaccharide (LPS) (1 µg/mL) were displayed by bar graph. Data were calculated and expressed as the mean ± SD; significance was calculated by paired *t*-test between the groups; ** *p* < 0.01, and *** *p* < 0.001. (**F**). Bar graph depicting percentage cytotoxicity by lactate dehydrogenase assay using supernatant of SIM-A9 cells treated with serum from CHOW, CHOW+GWI, WD, WD+GWI mice groups or with LPS (1 µg/mL), Data were calculated and expressed as the mean ± SD (*n* = 3). CD40, Cluster of Differentiation 40; TMEM119, Transmembrane protein 119; Il-6, Interleukin 6; DAPI, 4′,6-diamidino-2-phenylindole.

The data described above clearly show an association with microbiome dysbiosis in mice that are obese and have GWI but does not causally link the dysbiosis-induced systemic inflammation to changes in microglia. We determined whether the GWI and obesity-induced microbiome dysbiosis would release the circulatory mediators of microglial activation through an ex vivo/in vitro approach. SIM-A9 cells were incubated with mouse serum from the Chow, Chow+GWI, WD, and WD+GWI groups. An LPS-treated group was used as a positive control. Results showed that there was a significant increase in co-localization events of CD40 and TMEM119 in SIM-A9 cells-incubated with serum from the WD+GWI group when compared to the Chow or WD or Chow+GWI groups (Figure 10A,C) (*p* < 0.05). Tyrosyl radical formation, as indicated by 3-nitrotyrosine, significantly increased in the WD+Chow group when compared to the Chow or WD or Chow+GWI groups (Figure 10B,D) (*p* < 0.05). We examined whether the serum from mice with GWI and obesity would induce M1 polarization of microglia and release proinflammatory cytokine IL6, a crucial mediator in worsening GWI-associated neuroinflammation, by screening supernatants for the cytokine concentration using ELISA. Results showed that there was a significant increase in secreted IL6 in the supernatant of cells incubated with the WD+GWI group when compared to either the Chow or GWI alone or Chow+GWI groups (Figure 10E) (*p* < 0.01). The same group showed marked cytotoxicity as assessed by LDH release (Figure 10F). The results suggested that mouse serum from the WD-GWI group might harbor damage-associated molecular patterns, preferably HMGB1 or IL6, that may be instrumental in the activation of microglia shown in Figure 9.

## 4. Discussion

The present study describes the obesity-induced worsening of GWI-associated inflammation in the gastrointestinal tract, hepatic inflammatory changes that are a precursor to metabolic disturbances and neuroinflammation that may form the basis of cognitive decline and memory loss. The mouse model of chronic GWI was adopted from a published study by Zakirova Z et al. [45] to mimic exposures experienced by GW veterans who returned to a non-combat role after deployment and live in the United States [46]. In addition, like the rest of the continental US, veterans have adapted to a sedentary lifestyle or were physically incapacitated due to an injury suffered during the deployment [7]. This study used a Western diet (43% carbohydrate, 44% fat) that is a staple dietary pattern in the US and other Western societies [47]. Such a diet induces obesity and primarily mimics the present-day US dietary habit [48,49]. The Western diet remains a better model than a high-fat diet (60% kcal fat/low carb)-induced obesity for our studies since it models our veterans and is not merely mimicking a high-fat diet-induced morbidly obese condition with implications in the liver and other cardiovascular complications [50]. Our results show that obesity-induced by a Western diet for 20 weeks (~equivalent to 15.5 years in GW veterans) [51] resulted in a unique microbiome signature that caused a significant decrease in the butyrogenic bacterial species. Notably, the microbiome species changed in richness, diversity, and abundance, which was strongly correlated with gastrointestinal and hepatic alterations, and neuroinflammation.

To date, the current study is the most comprehensive microbial analysis in the GWI mouse model. The present study expands our earlier acute phase study that showed the microbiome dysbiosis via a V3–V4 16s sequencing [52]. With a rapidly evolving technology to evaluate microbiome dysbiosis, it is important that better and more sophisticated techniques must be applied to unearth the microbiome puzzle that may have lasting answers to the cause and the treatment of GWI. We used the shotgun metagenomics method for microbiome analysis at the species level using the next-generation sequencing platform, a sophisticated and better alternative to our earlier approach. A recent study reported the high-fat and low-carbohydrate diet (45% kcal fat)-induced microbiome changes in GWI, but the study was entirely based on 16s RNA sequencing. Though 16S rRNA gene sequencing is highly useful regarding bacterial classification, it may be noted that it has low phylogenetic power at the species level and a weak discriminatory power for some genera. Further, DNA-related studies are necessary to provide absolute resolution to these taxonomic problems [53,54,55].

*Akkermansia muciniphila*, a widely studied species known to have a beneficial effect in gastrointestinal disorders, was significantly decreased in Chow+GWI, and the decrease was strongly associated with gut abnormalities and inflammation [11,41]. In contrast, we observed a marked increase in abundance of *Akkermansia muciniphila* in the WD+GWI group (Supplementary Figure S1) when compared to the Chow+GWI group, suggesting that mucin degradation by this species was an adaptive response by the intestinal microenvironment to ensure that a proper defense can be launched following a possible rapid loss of membrane integrity in the WD+GWI group (increased Claudin-2 levels and decreased Occludin) though more detailed studies need to be performed to back the above hypothesis [11] (Figure 4A,B). It may be noted that *Akkermansia* species have been associated with intestinal membrane integrity [56]. However, the mechanisms of such a response are unclear at this time, and more species-level studies with higher n values need to be carried out to understand the commensalism of other species. The above argument is backed by our data related to the abundance of another beneficial microbe, *Lactococcus lactis*. *Lactococcus lactis* was found to be abundant in the WD+GWI group when compared to the Chow+GWI group. Interestingly, *Lactobacillus lactis* has been shown to release antioxidant defense enzyme superoxide dismutase and can be a strong player in the host anti-inflammatory response [42]. Together with *Akkermansia*, species such as *Lactobacillus lactis* may be aiding the host intestinal microenvironment to launch an effective and robust wound healing response following the chronic obesity-induced worsening of GWI intestinal inflammation (Figure 2B and Supplementary Figure S1). These findings are relevant for planning a future effective probiotic therapy where a combination of *Lactobacillus lactis* and *Akkermansia* sp. may be used in our GWI veterans.

We have shown before that butyrate-producing bacteria and bacterial species that promote gut health are significantly decreased in GWI. Similar results were also observed in the present study, where several butyrogenic bacteria and other prominent beneficial microbes were significantly decreased in the WD+GWI group (Figure 2E,F). Further, butyrate generated as a metabolic product of these bacteria has been shown to possess a robust anti-inflammatory effect largely by its ability to trigger an adaptive immune response involving T regulatory cells [57]. We used LEfSe analysis to determine specific bacteria taxa that were differentially abundant and partially depleted in the WD+GWI group. We identified *Hungatella* sp, *Eubacterium* sp, *Lachnospiraceae bact 3.1*, *Ruminococcaceae bact D16*, *Lachnospiraceae bact A2*, *Blautia*, and *Enterohabdus cecimuris* as the most differentially depleted genera associated with GWI-led worsening of symptoms in an underlying obesity condition (Figure 2B, Supplementary Figure S1). Notably, *Dorea* sp. decreased as well in the WD+GWI group, suggesting an adaptive response probably similar to the type of response we observed with *Akkermansia* sp. The results assume immense significance, since chronicity of GWI symptoms and their subsequent worsening might be due to the partial depletion of these beneficial microbes. However, gnotobiotic studies using the individual strains or a combination of these strains in the future will be valuable to authenticate these results. 

Chronicity of GWI manifests in mild to severe gastrointestinal disturbances in the subsection of patients [46]. We have shown that GI inflammation persists in the mouse model (Neu Insights-in press). The levels of proinflammatory events such as increased proinflammatory mediators like IL6, IL1β, inflammasome activation with concomitant alterations of tight junction proteins are strongly associated with persistent dysbiosis even 5 months after the GW chemical exposure that mimics war theater [11]. Our results of a further worsening of the GI inflammatory surge and events that contribute to a leaky gut in underlying obesity suggest that a Western dietary behavior and sedentary lifestyle (mice in the present model were not subject to exercise) can aggravate GI disturbances. Interestingly, one of our earlier studies showed an underlying liver inflammation but failed to show a liver disease phenotype that manifests in a significant elevation of liver enzymes or marked steatosis [44]. The underlying obesity due to prolonged Western diet consumption significantly elevated liver injury, as shown by increased Kupffer cell and stellate cell activation, markers of ensuing steatohepatitis, and or metabolic syndrome. The results and our assumptions about a possible metabolic syndrome were also backed by significant hyperinsulinemia and a decrease in insulin receptor substrate 1 level in the liver (Figure 6). Significantly enough, all the results associated with a diseased liver were strongly associated with an altered microbiome species diversity (Figure 5 and Figure 6). 

Gulf War illness veterans show a consistent decrement of brain function that includes cognitive and memory deficits [46]. Many of the symptoms reported by veterans with GWI are indicative of central nervous system (CNS) dysfunction, and indeed this has been corroborated by structural and functional neuroimaging and biomarker studies [46,58]. The decrements in brain function, such as memory and learning deficits, plague the veterans even today and highlight the need for proper diagnostic pathology and treatment. Most studies that model persistence of GWI symptoms do not consider the various comorbidities accompanying brain dysfunction in veterans. For example, a GWI veteran in the age range of 50–55 years and consuming a Western diet has limited mobility due to combat-related injuries, and may suffer from increased weight gain, hypertension, type II diabetes and a host of other chronic diseases. Thus, it is essential to study the weight gain-associated comorbidities and their role in worsening the neuronal disturbances. Our results of increased frontal cortex levels of IL6 and IL1β and their strong association with an altered species diversity show that obesity-induced unique microbiome signature might exacerbate the proinflammatory microenvironment in the brain. Interestingly, we have shown that IL6 remains elevated in serum and the brain following GW chemical exposure. IL6 and IL1β have been shown to contribute to major depression, dementia, and neurodegenerative disease phenotypes by [59,60,61,62]. Our results of a gut–brain connection to neuroinflammation strongly support the argument of increased focus on the mechanisms that may lead to these phenotypes, especially the role of microglial activation following stimulation by circulatory IL1β and IL6 [63]. Microglial activation in our mouse model showed a significant increase in the WD+GWI group when compared to the Chow+GWI group, suggesting that obesity-associated microbial dysbiosis and increased serum IL6 levels might contribute to such activation and possibly connect to neurodegenerative phenotype (Figure 8). Our results of exacerbation of neuroinflammation following Western diet-induced obesity are corroborated by other experimental studies where microglial activation is seen as an important event [64]. Microglial activation and subsequent amplification of inflammation in the brain is associated with regulation by neurotrophic factors such as BDNF [65]. Furthermore, obesity is associated with decreased levels of BDNF [66]. Further, the accumulation of phosphorylated tau is indicative of neurodegenerative changes [67,68]. The underlying obesity in the GWI mouse model resulted in a significant decrease in BDNF and a parallel increase in phosphorylated tau protein, indicating a neurodegenerative phenotype associated with microbial dysbiosis (Figure 9). Interestingly, microglial activation also leads to a neurodegenerative phenotype [69] and is extensively reviewed [70]. To strengthen our conclusion of such an association between dysbiosis, increased microglial activation and neurodegeneration, we incubated a transformed microglial cell line with serum from WD+GWI that had significant species diversity and a unique phenotype of increased bacterial species known to increase inflammation. Increased secretion of IL6, peroxynitrite generation, and increased cytotoxicity in these cells show a linear relationship between Western diet consumption, microbial dysbiosis, and neuroinflammation via a microglial activation pathway. Having shown that GWI mice with dysbiosis have an elevated circulatory IL6, it is not surprising that an even higher serum IL6 (Figure 3) in the WD+GWI group caused an M1 polarization (secreted IL6) to seeded microglial cells (Figure 10) [71]. The ex vivo/in vitro data also form the basis of a likely mechanism of microglia-induced neuroinflammation and neuronal dysfunction in the WD+GWI group. We also found a robust association between obesity in a GWI model and dysbiosis that is in line with a recent study in a Gulf War illness mouse model that showed dysbiosis following high-fat diet feeding [9]. Such a scenario is likely in GWI veterans because they are aging and have limited physical activity. Moreover, studies on the effects of mouse serum from the experimental in vivo groups exhibiting gut dysbiosis on microglia partially proved a relationship between gut dysbiosis and neuroinflammation. Our present study also advances our understanding about a likely scenario in veterans with an overweight or obese phenotype. Notably, one strength of our study lies in possible mechanistic links of an obese phenotype to persistence of GWI symptoms such as GI disturbances, liver abnormalities and neuronal pathology 28 years after the war ended. Nonetheless, a detailed mechanistic study using either an antibiotic-induced gut sterility control group or germ-free mice will be necessary to confirm the mechanisms mentioned above. Limitations include a moderate sample size of 5–6 mice per group and future studies should build on the preliminary findings of this study to focus on specific mechanistic underpinnings in each organ system with a large sample size. Further, GWI is a multisymptom disorder and dysbiosis-linked associations in pathology seen in mouse models only reflect a trend in disease progression rather than a causality. Improved rodent preclinical models may see benefits in finding definitive pathology and drug pathways. In summary, the results of this study suggest that the underlying obesity due to a Western diet contributes to persistence of gastrointestinal and hepatobiliary alterations and neuroinflammation over the longer term. Such effects are likely the result of a sustained microbial species-level diversity. Targeted probiotic approaches in combination with anti-inflammatory nutraceuticals might help to prevent a poor outcome in obesity-associated worsening of GWI symptoms that we observe in GW veterans today. Additionally, the results have implications for understanding the inflammation processes in chronic illnesses and the aging process.

## Supplementary Materials

The following are available online at https://www.mdpi.com/2072-6643/12/9/2764/s1, Figure S1: Differential abundance (LEfSe analysis) plot of significant OUT at species level between CHOW+GWI (GWI persistence in lean mice) and WD+GWI (GWI persistence in obese mice) Groups. The LEfSe analysis is calculated using three methods: Kruskal-Wallis sum-rank test, Wilcoxin rank-sum test, and Liniear Discriminant Analysis(LDA) meet *p* ≤ 0.05 for Kruskal-Wallis and Wilcoxin tests and have an LDA score ≥2.0 or ≤−2.0.
