# Supplementary material for: Obesity Worsens Gulf War Illness Symptom Persistence Pathology by Linking Altered Gut Microbiome Species to Long-Term Gastrointestinal, Hepatic, and Neuronal Inflammation in a Mouse Model
Source: Nutrients. 2020 Sep 10;12(9):2764. doi: 10.3390/nu12092764 (PMC7551189; doi:10.3390/nu12092764)
Supplement: Supplementary file 1 [file nutrients-12-02764-s001.zip › nutrients-903156-supplementary.pptx]

## Slide 1
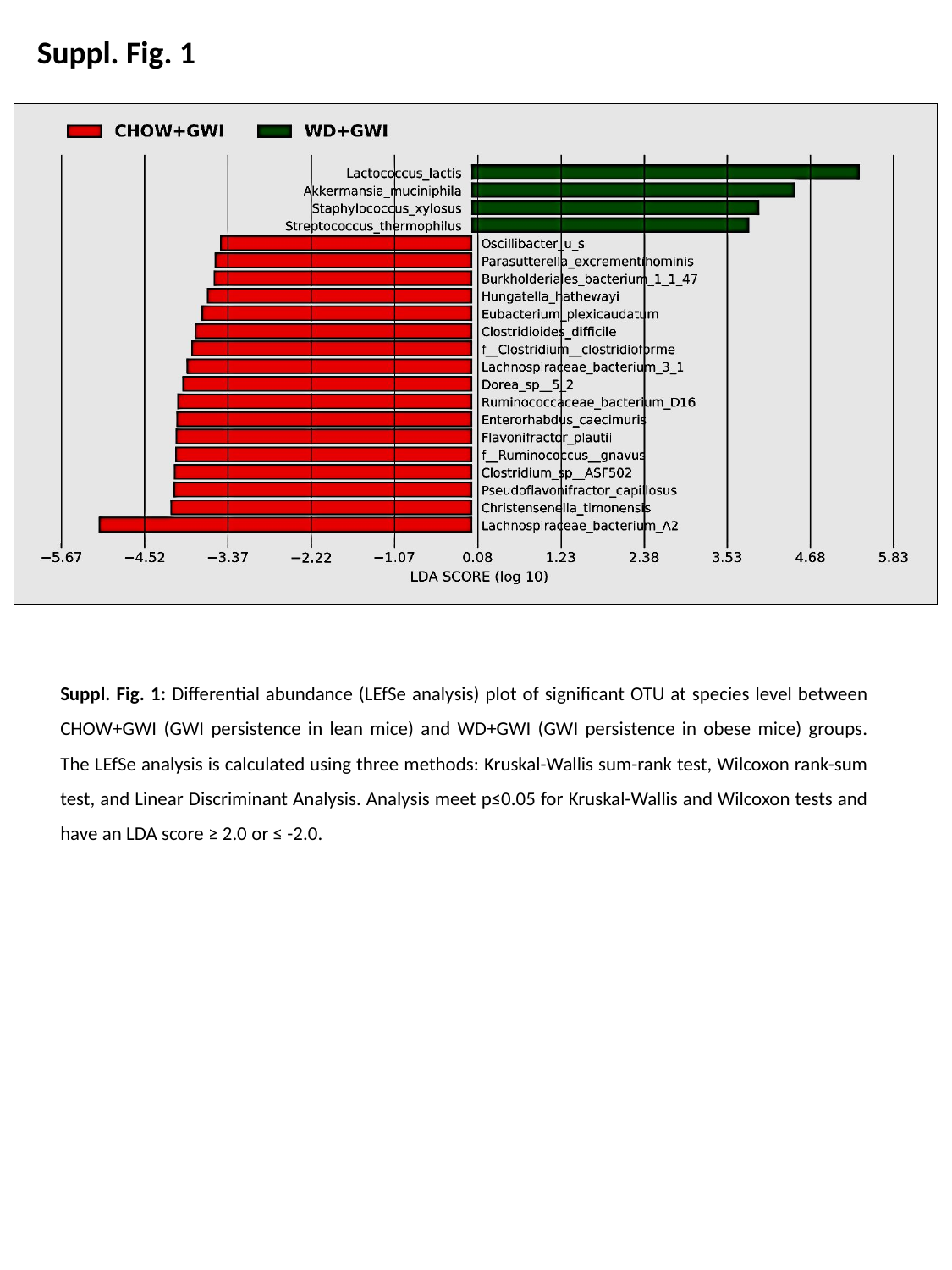

Suppl. Fig. 1
Suppl. Fig. 1: Differential abundance (LEfSe analysis) plot of significant OTU at species level between CHOW+GWI (GWI persistence in lean mice) and WD+GWI (GWI persistence in obese mice) groups. The LEfSe analysis is calculated using three methods: Kruskal-Wallis sum-rank test, Wilcoxon rank-sum test, and Linear Discriminant Analysis. Analysis meet p≤0.05 for Kruskal-Wallis and Wilcoxon tests and have an LDA score ≥ 2.0 or ≤ -2.0.
